# Study of epidemiological behaviour of malaria and its control in the Purulia district of West Bengal, India (2016–2020)

**DOI:** 10.1038/s41598-021-04399-x

**Published:** 2022-01-12

**Authors:** Sayantan Pradhan, Samrat Hore, Suman Kumar Maji, Simi Manna, Abhijit Maity, Pratip Kumar Kundu, Krishna Maity, Stabak Roy, Saptarshi Mitra, Paulami Dam, Rittick Mondal, Suvankar Ghorai, Junaid Jibran Jawed, Subhadeep Dutta, Sandip Das, Sukhendu Mandal, Sanjib Mandal, Ahmet Kati, Sangram Sinha, Amit Bikram Maity, Tuphan Kanti Dolai, Amit Kumar Mandal, İkbal Agah İnce

**Affiliations:** 1 Chemical Biology Laboratory, Department of Sericulture, Raiganj University, North Dinajpur, West Bengal 733134 India; 2 Hematology Department, Nil Ratan Sircar Medical College & Hospital, Kolkata, 700014 India; 3 Department of Statistics, Tripura University, Agartala, Tripura 799022 India; 4 District Public Health Centre, Deben Mahata Government Medical College and Hospital, Purulia, West Bengal 723101 India; 5 Department of Bio-Medical Laboratory Science & Management, Vidyasagar University, Midnapore, West Bengal 721102 India; 6 Calcutta School of Tropical Medicine, College Square, Kolkata, West Bengal 700073 India; 7 Department of Statistics, VisvaBharati University, Bolpur, West Bengal 731204 India; 8 Department of Geography and Disaster Management, Tripura University, Agartala, Tripura 799022 India; 9 Department of Microbiology, Raiganj University, North Dinajpur, West Bengal 733134 India; 10 School of Biotechnology, Presidency University – 2nd Campus, Kolkata, West Bengal 700156 India; 11 Department of Botany, School of Sciences, Durgapur Regional Centre, Netaji Subhas Open University, West Burdwan, Kolkata, West Bengal 713214 India; 12 Laboratory of Molecular Bacteriology, Department of Microbiology, University of Calcutta, Kolkata, 700019 India; 13 Department of Economics, Raiganj University, North Dinajpur, West Bengal 733134 India; 14 Department of Biotechnology, Institution of Health Sciences, University of Health Sciences, Uskudar, Istanbul, 34668 Turkey; 15 Department of Botany, Vivekananda Mahavidyalaya, Haripal, Hoogly, West Bengal 712405 India; 16 Department of Otorhinolaryngology, Deben Mahata Government Medical College and Hospital, Purulia, West Bengal 723101 India; 17 Centre for Nanotechnology Sciences, Raiganj University, North Dinajpur, West Bengal 733134 India; 18 Department of Medical Microbiology, School of Medicine, Acibadem Mehmet Ali Aydınlar University, Ataşehir, Istanbul, 34752 Turkey

**Keywords:** Diseases, Health care

## Abstract

Purulia is a malaria-prone district in West Bengal, India, with approximately half of the blocks defined as malaria endemic. We analyzed the malaria case in each block of the Purulia district from January 1, 2016, to December 31, 2020. As per the API, 20 blocks of Purulia were assigned to four different categories (0–3) and mapped using ArcGIS software. An exponential decay model was fitted to forecast the trend of malaria cases for each block of Purulia (2021–2025). There was a sharp decrease in total malaria cases and API from 2016 to 2020 due to the mass distribution of LLINs. The majority of cases (72.63%) were found in ≥ 15-year age group. Males were more prone to malaria (60.09%). Malaria was highly prevalent among Scheduled Tribes (48.44%). Six blocks were reported in Category 3 (high risk) and none in Category 0 (no risk) in 2016, while no blocks were determined to be in Category 3, and three blocks were in Category 0 in 2020. The exponential decay model prediction is oriented towards gaining malaria-free status in thirteen blocks of Purulia by 2025. This study will incite the government to uphold and strengthen the current efforts to meet the malaria elimination goals.

## Introduction

Malaria, a mosquito-borne infectious disease caused by unicellular eukaryotic *Plasmodium* parasites, is one of the most critical public health issues globally. Five different types of *Plasmodium* parasite species cause human malaria: *P. falciparum (Pf)*, *P. vivax (Pv)*, *P. malariae*, *P. ovale*, and *P. knowlesi*, transmitted by female *Anopheles* mosquitoes^1^.

In 2019, 229 million malaria cases were reported globally in 87 malaria-endemic countries by the World Health Organization (WHO). In 2000, 238 million malaria cases were estimated. Globally, 29 countries accounted for 95% of malaria cases, with only five countries accounting for more than half of global malaria cases (e.g., Nigeria: 27%, the Democratic Republic of the Congo: 12%, Uganda: 5%, Mozambique: 4%, and Niger: 3%). In 2019, Africa witnessed approximately 94% (215 million) of worldwide malaria cases, with the South-East Asia region reporting 3% (6.3 million) of malaria cases. In this region, India reduced malaria cases from approximately 20 million in 2000 to 5.6 million in 2019 while Sri Lanka achieved malaria-free certification in 2015. Zero malaria cases were reported in Timor-Leste in 2018 and 2019. According to the WHO, 5 million, 1.7 million, and 0.9 million malaria cases were reported in the Eastern Mediterranean, Western Pacific, and America’s regions, respectively, in 2019. The WHO European Region was proclaimed malaria-free since2015. In 2019, 409,000 malaria deaths were estimated globally, and 31 countries accounted for approximately 95% of malaria deaths among these cases. Six countries witnessed more than half of global malaria deaths (Nigeria: 23%, the Democratic Republic of the Congo: 11%, the United Republic of Tanzania: 5%, Mozambique: 4%, Niger: 4%, and Burkina Faso: 4%). Approximately 384,000 malaria deaths were reported in the WHO African Region in 2019, and 9,000 were reported in the WHO South-East Asia Region in the same year. In the WHO South-East Asia Region, around 86% of malaria deaths were reported from India. In 2019, the WHO reported 10,100, 3200, and 551 deaths in the Eastern Mediterranean Region, the WHO Western Pacific Region, and the Region of Americas, respectively^2^.

In 2019 and 2020, malaria cases were predominant in five states of India: Uttar Pradesh, Chhattisgarh, Orissa, Jharkhand, and West Bengal (NVBDCP)^3^. Malaria infection is a critical public health crisis in rural or tribal areas of India, particularly in sixteen states, including seven northeastern and nine central states^4, 5^. An increased malaria burden has been experienced by tribal communities in these states, especially those living in remote areas due to expansive geographical features such as dense forests, valleys, hills, and perennial streams. The diverse climate of this region favors the growth and proliferation of malaria parasites and vector species contributing to the transmission of malaria^6^. Data confirm that malaria has been endemic in the Purulia district of West Bengal, India, for the past few decades^7^. Despite the high incidence of malaria in this district, research on malaria epidemics is limited due to the lack of research infrastructure and the region’s remote location and inaccessible terrain. For these reasons, there is insufficient data available for the proper prediction and management of malaria cases.

The Global Fund partnership (https://www.theglobalfund.org/en/) is designed to accelerate the worldwide fight against AIDS, tuberculosis, and malaria. In India, the Global Fund for malaria has been approved to support the National Vector Borne Disease Control Programme (NVBDCP), the Ministry of Health & Family Welfare, and the Government of India. NVBDCP aims to eliminate malaria throughout the country (e.g., achieve zero indigenous cases) by 2030 and maintain malaria-free status in regions where malaria transmission has been lowered or eliminated^8^. Reduced morbidity and mortality from malaria are mainly attributed to improved vector control measures, such as providing Long-Lasting Insecticidal Nets (LLINs) to residents. Furthermore, blood collection [active collection, passive collection, fever & contact (survey) collection, and mass collection] for microscopic examination, early diagnosis, and treatment are procured to monitor, examine and counter each malaria case throughout the Purulia District. As stated by National Vector Borne Disease Control Programme (NVBDCP) and World Health Organization (WHO) guidelines, rapid diagnostic test (RDT) kits allow early detection of plasmodial antigens making the surveillance system robust through prompt diagnosis and treatment initiation^8, 9^. Treatment approaches e.g., Artemisinin-based combination therapies (ACTs) have been deployed while imposing a countrywide withdrawal of monotherapy using oral artemisinin for preserving its efficacy^9–12^. The Department of Health, the Government of West Bengal distributed the supplied LLINs by the NVBDCP and the Government of India to 100% of the households of malaria risk population in ten malaria-endemic blocks identified from the 2016 Annual Parasite Index (API) of Purulia district, as a measure to protect the residents from mosquito bites and reduce transmission^13–16^. API is an estimate of malaria morbidity of any geographical level for a given year. It is determined as the number of malaria-positive patients per 1000 inhabitants (Total no. of positive slides/Total no. of slides × 1000).

This is the first detailed report on the epidemiological study of malaria, including all blocks in Purulia district, West Bengal, India, to the best of our knowledge. The aim of the study was to classify all blocks of the Purulia district into four different categories as per API followed by geographic information system (GIS) mapping to identify malaria prone blocks from 2016 to 2020 and to provide the retrospective trend of space–time distribution of malaria cases in the district. The effect of LLINs mass distribution was monitored via measuring malaria cases before and after the campaign. Nevertheless, our aim was to develop a prediction model that could determine the impact of various government strategies opted for malaria reduction and refine future policy-making.

Due to the ongoing global SARS-CoV-2 pandemic, it is believed that patients affected with malaria may postpone or avoid seeking proper treatment from the established health care facilities^17, 18^. Thus, strategies aimed at controlling mosquitoes and reducing malarial infection rates are extremely critical at this time.

## Methods

### Study area

The Purulia district (22°–60′ 23°–50′ N and 85°–75°′–86°–65′ E) is one of twenty-three districts in the State of West Bengal in India. The total population of Purulia is 3,039,583^19^. Sharing a border with Jharkhand, the district encompasses 6259 Sq. kms (Supplementary Fig. S1). Average annual rainfall is approximately 1268 mm, and average daily temperature ranges from 6 in winter to 46 °C in summer, with high relative humidity during the monsoon season ranging from 75 to 85%^20^. In total, the Purulia district consists of 20 blocks. Of these, half are considered to be malaria-endemic, according to the 2016 API. The majority of the 20 blocks are surrounded by inaccessible terrain.

### Case detection

Two tests are currently available for detecting malaria that meets the guidelines of the NVBDCP and the Government of India: Rapid Diagnostic Test (RDT) and Microscopy. While the RDT provides rapid early diagnosis within twenty minutes, Microscopy is regarded as the gold standard for confirming the presence of malaria parasites^12, 21, 22^. The RDT has enabled more accessible early detection and treatment in hard-to-reach areas and made data more reliable and easier to collect to monitor morbidity and mortality associated with malaria. In tandem with microscopy-based techniques, the RDT is used to monitor the effectiveness of malaria treatments and aid in administering the anti-malarial drug (ACT) viz. Artesunate + sulfadoxine-pyrimethamine (SP), Artemether + lumefantrine, Artesunate + amodiaquine, etc^9^. In this study, both the RDT and Microscopy techniques were included to diagnose malaria cases.

### Data collection

This epidemiological study examined malaria cases in all 20 blocks of the Purulia district between January 1, 2016, and December 31, 2020. Data were collected from Block Primary Health Center’s (BPHCs) laboratories, Primary Health Center’s (PHCs), different sub-centers, District Hospital, Sub-divisional Hospital, and malaria sentinel fields. Annual reports of district-level aggregated malaria case data were also collected from the Department of Health and Family Welfare, Purulia District. Collected data included species type distribution, age-sex distribution, seasonal variation, and caste distribution. The information regarding LLINs distribution was obtained from Zilla Swasthya Bhawan, Purulia, for the ten endemic blocks that received these resources in mid-2017 and 2018^13^.

### The GIS analysis

As per API in 2016, all blocks of the Purulia district were categorized and mapped by high-resolution GIS^23, 24^. These maps overlaid with API details served as a practical resource for planning malaria control, implementing various programs, and taking initiatives to monitor malaria cases. As per API, twenty blocks of the Purulia district were assigned to four categories, i.e., category 3, 2, 1, and 0 (Category-3: The total block API & also minimum any one or more than one sub-center API requires being ≥ 1 case per 1000 population at risk, Category-2: The entire block API requires being < 1 case per 1000 population at risk, but minimum one sub-center API should be ≥ 1 case per 1000 population at risk, Category-1: The total block API & also all sub-center API requires being < 1 case per 1000 population at risk, and Category-0: The block with 0 malaria case) from 2016 to 2020 and mapped using ArcGIS software version 10.8^13, 23, 24^.

### Data analysis

Data analysis of age-sex and caste distribution of malaria cases was performed using the “R” statistical software (version 3.4.1). Season-wise change patterns of the malaria cases over different months of the 5-year study period were reported in Supplementary Table S6. The effectiveness of LLINs distribution on malaria cases of ten endemic blocks was graphed using Microsoft Excel. χ^2^ tests were carried out to determine whether there were differences in cases among castes over time or differences in cases between genders in different age groups. The significance level was set at < 0.05. An exponential decay model was also fitted for the available data set and used to project the malaria cases for every block of the Purulia district for the next 5 years, up to 2025. Heat maps were generated through Microsoft Excel 2013 to investigate the distribution of malaria cases in Purulia District.

### Ethics declaration

This study was permitted by Zilla Swasthya Bhawan, Purulia, Govt. of West Bengal (Memo No. 2041; Dt. 03.11.2020). The ethical approval (No. RUHECRP001) was obtained from the Raiganj University Human Ethical Committee (RUHEC). The data were anonymized and combined at the Zilla Swasthya Bhawan without any individual patient information. All procedures were executed according to the pertinent guidelines and regulations. Informed consent was collected from all participants or if participants are under 18, from a parent and/or legal guardian.

## Results

### Species type distribution of malaria cases

There were 5849 confirmed malaria cases in the twenty blocks of the Purulia district during the study period between January 1, 2016, and December 31, 2020 (Supplementary Table S1). The total number of malaria cases decreased from 2864 cases in 2016 to 2033 in 2017 (29.02% reduction). *Pv* accounted for 1120 cases in 2016 and 809 cases in 2017 (27.77% reduction). *Pf* cases decreased from 1356 cases in 2016 to 1052 cases in 2017 (22.42% reduction) while mixed infection cases decreased from 388 cases in 2016 to 172 cases in 2017 (55.67% reduction). There was a remarkable decrease of 79.24% in total malaria cases from 2017 (2033) to 2018 (422): *Pv* cases dropped from 809 in 2017 to 197 in 2018 (75.65%), *Pf* cases from 1052 in 2017 to 181 in 2018 (82.79%), and mixed infection cases from 172 in 2017 to 44 in 2018 (74.42%). However, mixed trends were observed between 2018 and 2019. Total cases dropped from 422 cases in 2018 to 331 cases in 2019 (21.56%). *Pv* cases decreased from 197 cases in 2018 to 87 cases in 2019 (55.84%). *Pf* cases slightly increased from 181 cases in 2018 to 193 cases in 2019 (6.63%) and mixed infection cases also a bit increased from 44 cases in 2018 to 51 cases in 2019 (15.91%). In contrast, decreases were once again seen from 2019 to 2020, with total malaria cases dropping from 331 to 199 (39.88%), *Pv* cases from 87 to 40 (54.02%), *Pf* cases from 193 to 131 (32.12%), and mixed infection cases from 51 to 28 (45.1%).

### Demographic summary of malaria cases

Table 1 represents the demographic breakdown of malaria cases in the Purulia district in the study period. Supplementary Figure S2 represents block-wise malaria risk zones of each year during the study period based on malaria cases. To determine the significant effect of gender and age-wise classifications on reported malaria cases during the 2016 to 2020 study period, the Chi-square test was carried out at a 5% significance level. Results showed that 3515 male (60.09%) and 2334 (39.91%) female patients were diagnosed with malaria. A χ^2^ test (χ^2^ = 69.4971, p < 0.05) indicated that there were significant differences among malaria cases by age groups and gender. Malaria cases were significantly higher (χ^2^ = 69.4228, p < 0.05) for people ≥ 15 years compared to the other two age groups, irrespective of gender, indicating ≥ 15 years age group is mostly affected by malaria. A similar result was found in an earlier study^25^.

**Table 1:** Demographic breakdown of malaria cases in Purulia district from 2016 to 2020.

Variable: classifications		Year					Marginal total
		2016	2017	2018	2019	2020	
Gender	Male	1752	1159	283	201	120	3515
	Female	1112	874	139	130	79	2334
Age	< 5 years	171	147	26	18	11	373
	5to < 15 years	598	443	79	65	43	1228
	≥ 15 years	2095	1443	317	248	145	4248
Caste	SC	248	168	38	42	11	507
	ST	1416	921	249	151	96	2833
	Others	1200	944	135	138	92	2509
Marginal total		2864	2033	422	331	199	**5849**

Significance value is given in bold.

Further analysis was performed to determine if there were any effects of caste [Scheduled Caste (SC), Scheduled Tribe (ST), and others] on the malaria cases in this region over the past five years. A total number of 507 cases (8.67% of the total malaria cases) were diagnosed for SC, 2833 cases (48.44%) for ST, and 2509 cases (42.89%) for other castes in a ratio of 1:5.6:4.9 (Supplementary Table S2). A higher number of cases were observed for the ST caste than was expected (χ^2^ = 42.79, p < 0.05).

### Maleriometric indicators

For all Purulia district blocks, the API indicator (out of 1000 inhabitants under surveillance) showed a gradual decrease and was found to be 1.0, 0.71, 0.15, 0.11, and 0.07 for 2016, 2017, 2018, 2019, and 2020, respectively. Similarly, the rate indicator i.e., Annual Blood Examination Rate (ABER calculated as: Smear examination in a year/estimated total population of areas at risk of malaria × 100) was 17.84% (2016), 18.52% (2017), 16.5% (2018), 17.07% (2019) and 12.70% (2020), respectively. Malariometrics indices from 2016 to 2020 are given in Table 2.

**Table 2:** Malariometricindices of Purulia district from 2016 to 2020.

Malariometric indicators	2016	2017	2018	2019	2020
	Population 2,867,839	Population 2,876,835	Population 2,894,114	Population 2,899,541	Population 2,908,583
Cases	2864	2033	422	331	199
API	1	0.71	0.15	0.11	0.07
ABER	17.84	18.52	16.5	17.07	12.70

[API (Annual parasitic incidence) = Total number of positive slides for parasite in a year × 1000/Total population.

ABER (Annual blood examination rate) = (Smear examination in a year × 100)/Total population.

### Categorization of all blocks according to API

**Figure 1:**
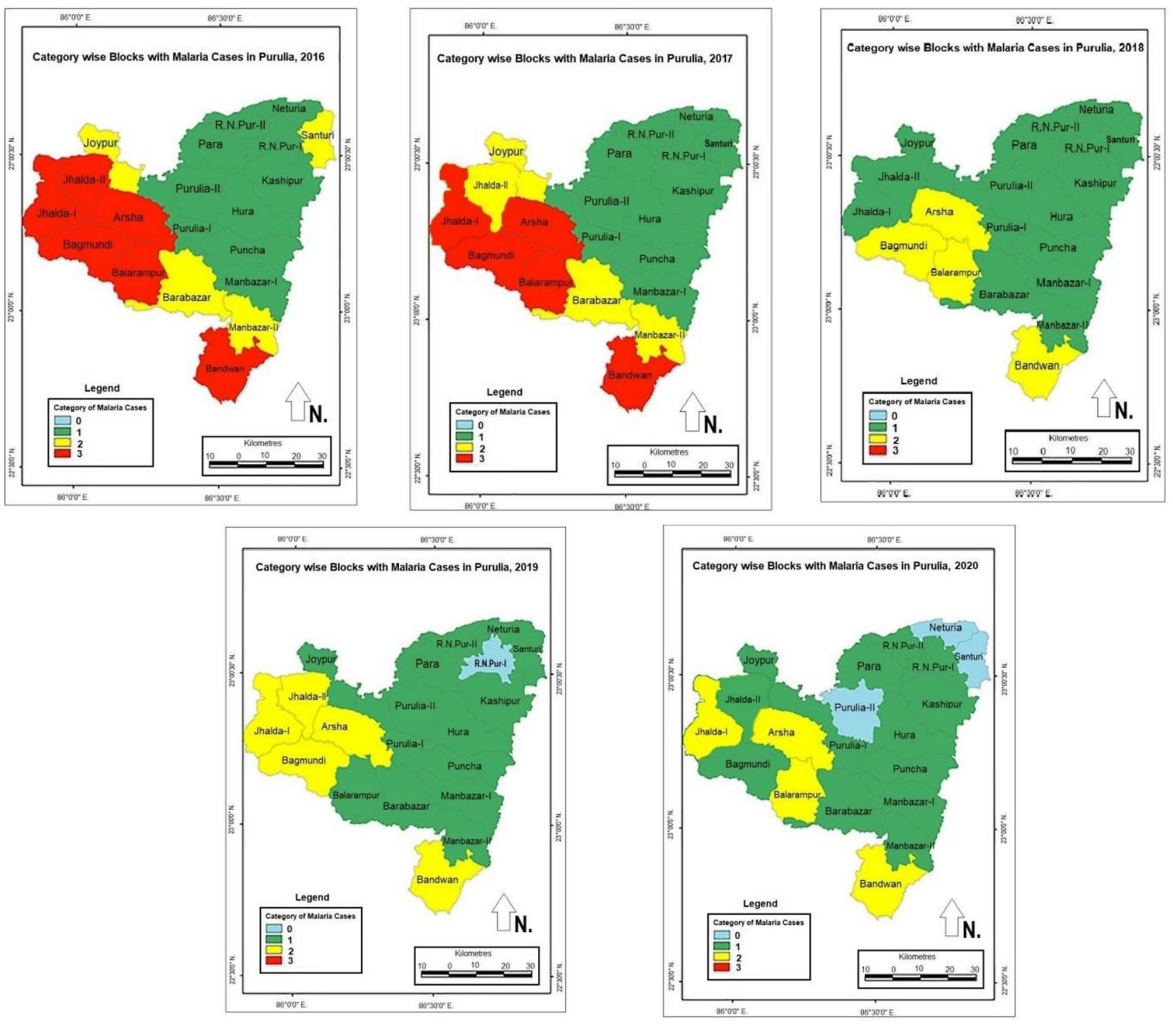
Categorization of blocks in Purulia district as per API criteria, from 2016 to 2020. The map has been prepared by using ArcGIS v.10.8.

Malaria cases were recorded in all Purulia district blocks from 2016 to 2020. The blocks were divided into four different categories by evaluating corresponding API criteria, as shown in Supplementary Table S3. Figure 1 highlights the trends followed by the blocks for each year, with block names in Supplementary Table S4 and block categories summarized by year in Supplementary Table S5. During this five-year study period (2016–2020), the malaria prone Purulia district appeared to begin recovery from this epidemic as fewer cases have been reported every year. According to the API, 6 and 5 blocks seemed to be the most affected (Category 3) in 2016 and 2017. However, for the last 3 years of the study, no block was classified as Category 3, suggesting improvements in infection rates. In 2016, the following blocks were classified as Category 3: Bandwan (1), Balarampur (5), Arsha (8), and Jhalda-I (14). However, by the end of this study in 2020, these blocks were all classified as Category 2. Additionally, Bagmundi (7) and Jhalda-II (13) were categorized in Category 1 in 2020 after being placed in Category 3 in 2016. Similarly, in 2016, Category 2 blocks included Manbazar-II (2), Barabazar (4), Joypur (16), and Santuri (19). By 2020, the first three blocks improved to Category 1, and zero cases (Category 0) were reported for the Santuri (19) block. Other blocks, including Manbazar-I (3), Puncha (6), Hura (9), Purulia-I (10), Kashipur (12), Para (15), and R.N. Pur-II (18), remained in the same category throughout the study (Category 1). However, significant improvements were observed for Purulia-II (11), Santuri (19), and Neturia (20), with zero cases reported (Category 0) in 2020. R.N. Pur-I (17) was the first reported block with zero cases in 2019; however, this trend did not continue to 2020.

### Seasonal variation of malaria cases

From 2016 to 2020, the maximum number of malaria cases in the Purulia district was reported in August, except for 2017 and 2020, where the maximum number of malaria cases was reported in July (Supplementary Table S6). The ultimate case numbers’ timing largely coincided with the rainy season (July–August). Interventions are required to raise public awareness of the high mosquito population during the rainy season and help implement preventative measures to reduce malaria cases.

### Heat map analysis of malaria cases (2016–2020)

**Figure 2:**
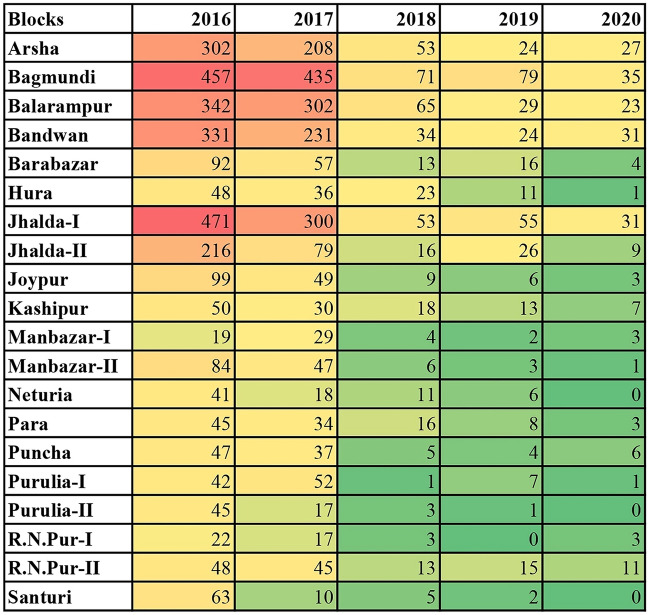
Heat map analysis of block-wise malaria cases in Purulia districtover the years 2016–2020. Color spectrum from red to green indicates progressive decrease in number of malaria cases. Green denotes the low number of cases; Red denotes high number of cases.

A heat map analysis of block-wise malaria cases in Purulia district over the years 2016–2020 was presented in Fig. 2, using a color scale ranging from green (low number of malaria cases) through yellow (medium number of malaria cases) to red (high number of malaria cases), over the years 2016–2020. For most blocks, malaria cases declined over time compared to 2016. In 2016, more than 200 cases were reported for six blocks (highlighted in red), while all remaining 14 blocks reported less than 100 cases highlighted in yellow. However, for the year 2020, only six blocks had more than ten reported cases, and the remaining 14 blocks had less than 10 cases. Among these, the Purulia-II, Santuri, and Neturia blocks had zero reported cases in 2020.

### LLINs distribution

According to the 2016 API, LLINs were distributed among ten endemic blocks of the Purulia district in 2017 and 2018. 181,012 LLINs were distributed among 325,334 residents in 2017, and 300,000 LLINs were distributed among the same number of residents in 2018. On average, one LLIN was provided for every 1.797 individuals (~ 2 individuals) in 2017 and every 1.084 individuals (~ 1 individual) in 2018 (Supplementary Table S7)**.**

### Malaria scenario of pre- and post LLINs distribution

From 2016 to 2020, malaria cases in these ten endemic blocks were calculated as 2457, 1718, 325, 264, and 164 in 2016, 2017, 2018, 2019, and 2020, respectively (Table 3). Overall, malaria cases decreased in these ten endemic blocks by 93.33% in 2020 after the mass distribution of LLINs. It is paramount that disease incidence either remains at this low level or continues to decline (Supplementary Fig. S3)**.**

**Table 3:** Number of malaria cases in 10 endemic blocks (before and after LLIN distribution) of Purulia district from 2016 to 2020.

Year	Malaria cases
2016	2457
2017	1718
2018	325
2019	264
2020	164

### Prediction of malaria cases through exponential decay model (2016–2025)

**Figure 3:**
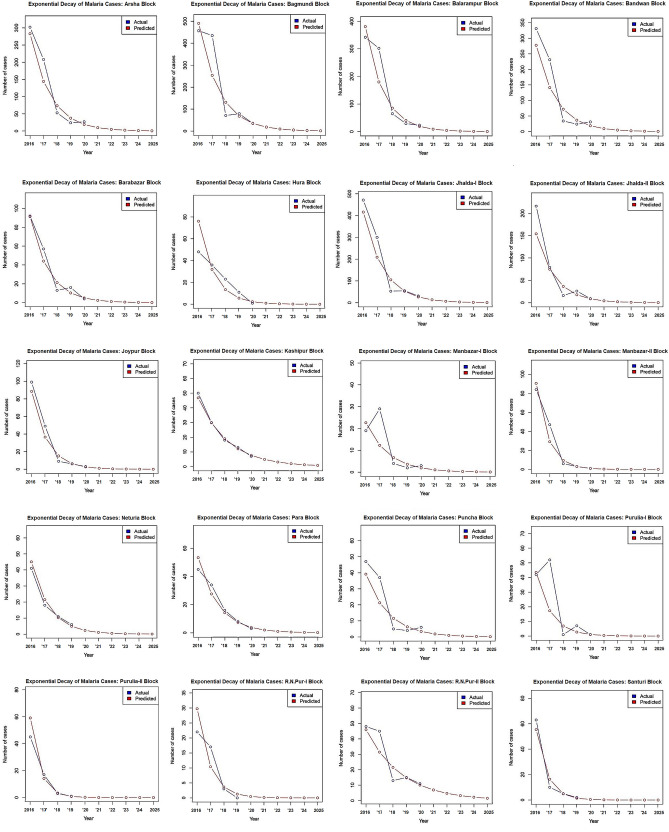
Predictions of malaria cases from 2016 to 2025 via exponential decay model analysis while assuming other factors (e.g., environmental factors etc.) are constant throughout the study period. These plots were created with “R” statistical software using version 3.4.1 (R Foundation for Statistical Computing, Vienna, Austria, 2017. https://www.R-project.org/).

Next, we forecasted the future event for each block of the Purulia district from 2020 to 2025. An exponential decay model was fitted based on the collected dataset from 2016 to 2020. The estimated number of cases for each block was reported in Table 4 and Fig. 3. Furthermore, the actual malaria cases from 2016 to 2020 versus predicted malaria cases by the exponential decay model from 2016 to 2025 are also presented in Fig. 3.

**Table 4:** Exponential decay model with estimated parameter values for each block.

SL. no.	Block	$f\left(t\right)= a{\mathrm{e}}^{\mathrm{bt}}$	SL. no.	Block	$f\left(t\right)= a{\mathrm{e}}^{\mathrm{bt}}$
1	Arsha	73.58e^−0.673t^	11	Manbazar-I	6.67e^−0.611t^
2	Bagmundi	131.30e^−0.659t^	12	Manbazar-II	9.34e^−1.136t^
3	Balarampur	85.15e^−0.749t^	13	Neturia	14.86e^−0.369t^
4	Bandwan	71.99e^−0.675t^	14	Para	14.25e^−0.661t^
5	Barabazar	21.28e^−0.729t^	15	Puncha	11.59e^−0.609t^
6	Hura	13.42e^−0.867t^	16	Purulia-I	6.87e^−0.923t^
7	Jhalda-I	105.01e^−0.688t^	17	Purulia-II	6.92e^−0.714t^
8	Jhalda-II	36.39e^−0.721t^	18	R.N.Pur-I	10.39e^−1.051t^
9	Joypur	15.10e^−0.884t^	19	R.N.Pur-II	21.54e^−0.379t^
10	Kashipur	18.97e^−0.451t^	20	Santuri	8.91e^−0.609t^

### Heat map analysis of projected malaria cases

**Figure 4:**
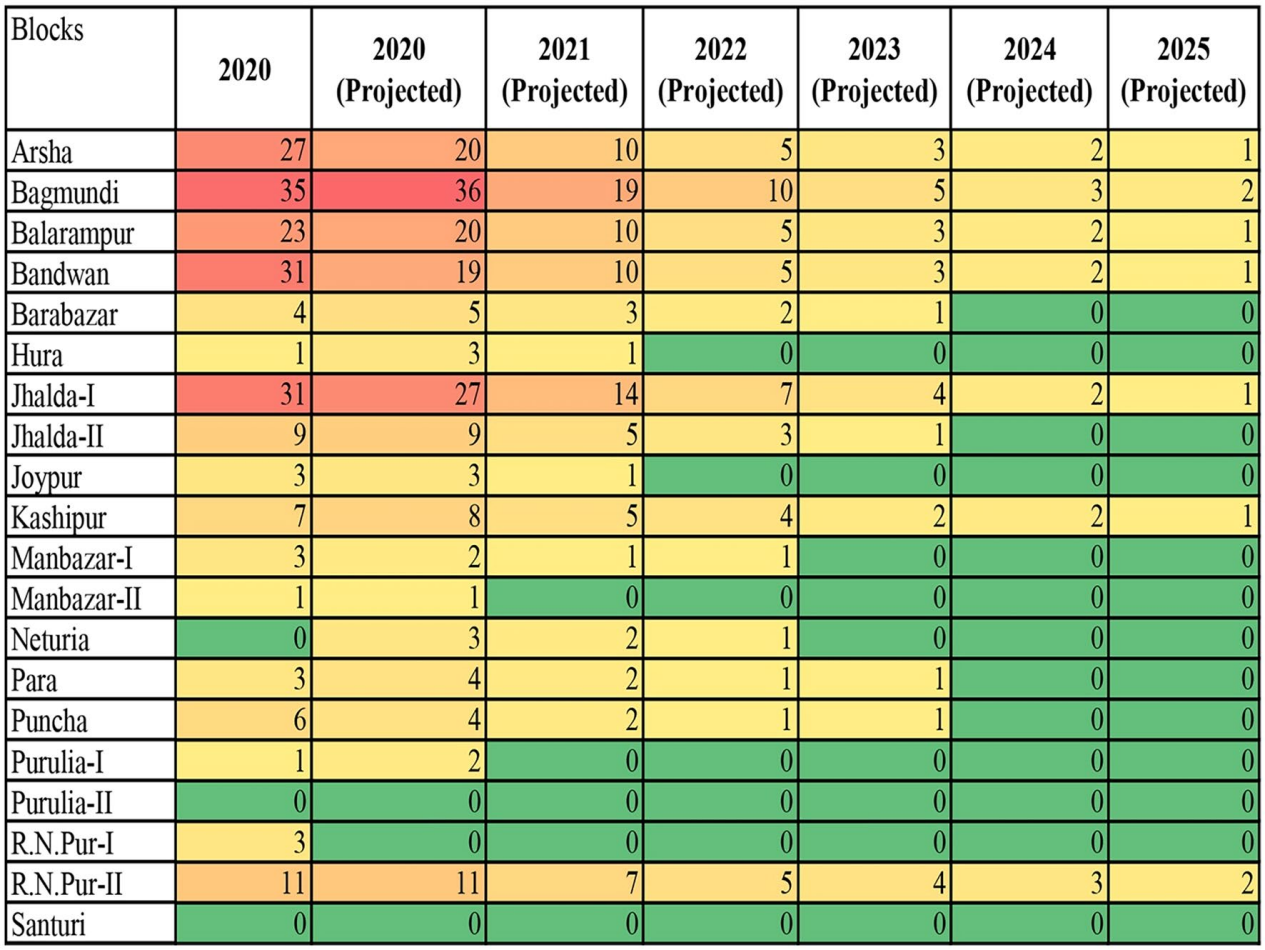
Heat map analysis of block-wise projected malaria cases in Purulia districtover the years 2020–2025. Color spectrum from red to green indicates progressive decrease in number of malaria cases. Green denotes the low number of cases; Red denotes high number of cases.

Forecasted future values through the exponential decay model were presented using heat-map analysis in Fig. 4. Although an overall trend of decreasing case values was detected for each block of the Purulia district, in some cases, the number of actual cases exceeded its predicted number of cases for 2020, including the blocks of Arsha, Balarampur, Bandwan, and Jhalda-I, which showed 35%, 15%, 63.16% and 14.81% more cases that had been predicted, respectively. A few blocks reported only one or two more cases than were projected. For example, R.N. Pur-I was predicted to be malaria-free in 2019, but three cases were reported in 2020. For a 5-year plan, Purulia may be a malaria-free district if proper actions are taken. Our results further suggested special attention is required to the blocks with steady levels of low case numbers, such as Kashipur and R.N. Pur-II.

## Discussion

Malaria indicators aim to offer epidemiological tools for generating, analyzing, and utilizing data on malaria quantification, distribution, and prioritizing the risk factors to allow the proper selection of intervention strategies for effective surveillance and control. The 20 blocks in the district of Purulia exhibit a significant variation in the incidence of malaria transmission and risk of infection (Fig. 1). The 2016 API was used to measure malaria cases and epidemiological effects in the Purulia district and stratify the malaria hotspots per risk level. The study contributes towards the evaluation and reorientation of action plans and public health policies directed towards malaria control. We have observed that the API was highest in 2016 and then decreased gradually (Table 2).

Malaria was found across all age groups in our study. Specifically, malaria cases were higher in individuals 15 years of age and older age group (n = 4248, or 72.63% of the total malaria cases). This higher level of cases may be linked to occupational activities as a high percentage of individuals in this age group participate in outdoor activities^26^. 27.37% of cases were reported among individuals less than 15 years of age. We also noted that malaria infection was more predominant in males (60.09%) than females, which could be related to their behavior. It was reported earlier that malaria transmission might be higher among those who report to their worksite during the early evening^27^.

From the data analyzed according to blocks in the Purulia district from 2016 to 2020, we observed that the prevalence of malaria was higher among the tribal populations than the scheduled caste and other caste populations (SC = 8.67%, ST = 48.44%). Numerous streams and their tributaries inundate the tribal villages of the Purulia district, which aid the reproduction of mosquitoes all through the year with a significant increase in malaria cases in the rainy season (Supplementary Table S6)^4, 28^. The battle against the rising burdens of malaria in the tribal belts demands the implementation of multi-dimensional approaches along with socio-economic progress among tribal people^4^.

In this study, GIS maps visually indicated malaria hotspots in the Purulia district in West Bengal from 2016 to 2020. Most malaria cases occurred in the remote blocks that are encompassed by forests and hilly regions. Based on the API of 2016, LLINs were distributed to the malaria risk population present in 10 endemic blocks of Purulia district in 2017 and 2018, assuming that the population would have stayed constant for both the years (Supplementary Table S7). The GIS maps also indicated a decreasing trend of malaria cases after the distribution of LLINs during these two years. Based on data and maps, feedback was given to decision-makers and local health staff to prioritize malaria control activities and strengthen malaria control capacity under limited financial and human resources.

The prevalence of malaria cases decreased in the Purulia district after the distribution of LLINs in 2017 and 2018 in the malaria risk population of ten endemic blocks. On average, the Purulia district provided one insecticide-treated net for two individuals in 2017 and one individual in 2018 in the ten malaria-endemic blocks, at a rate higher than many other malaria-endemic countries^29^. Notably, LLINs distribution may have a direct impact on malaria cases^30, 31^. For example, according to the 2016 API, the Santuri block (63 cases) was selected for LLINs distribution, whereas LLINs were not distributed in R.N. Pur-II (48 cases). A decreasing trend in malaria cases was detected in Santuri from 2017 onwards, and no cases were reported in 2020. In contrast, the malaria cases in R.N. Pur-II remained steady from 2017 to 2020, and this block may not be malaria-free in 2025, according to the projected exponential model (Fig. 4). Our study also had several limitations. First, the study was limited by a single malaria-endemic district restricting the actual scenario of malaria in West Bengal. Second, at the time of projection determination, we have assumed that different environmental factors like rainfall, aridity, humidity, precipitation and other factors are constant throughout this study period. However, there were confounding factors that could play a significant role in this projection. Finally, due to COVID-19 pandemic, the essential malaria interventions are significantly interrupted, causing difficulties in finding out the real number of malaria cases in this malaria-endemic district.

## Conclusion

In conclusion, this is the first detailed study in Purulia District, West Bengal, India, providing the overall illustration of malaria cases. The malaria hotspots of Purulia district were identified through GIS mapping, focusing on the improvement of the malaria surveillance system and scaling up the existing malaria treatment strategy. Our findings demonstrate a downward trend in malaria cases over the past 5 years. LLINs distribution among the inhabitants of some endemic blocks appears to have significantly reduced the number of malaria cases in these areas. LLINs distribution, coupled with well-designed information, education, and communication (IEC) approach among the inhabitants, may continue to reduce the number of cases further, eventually leading to eradicating malaria cases from Purulia. The ongoing and increasing obstacles in the worldwide struggle to eradicate malaria underscore the importance of healthcare professionals, malaria researchers, proper interventions, and the international financing community being steadfast in their efforts to eradicate the life-threatening disease. Our research findings may provide a significant resource for these communities, which will help them in decision making in the near future. Consistent funding is needed to avoid reappearance and sustain eradication goals in such malaria-endemic districts. Despite these limitations, this study is the first attempt to create a data-driven malaria predictor of malaria in the malaria-prone zone.

## Supplementary Information

Supplementary Information.

## Data Availability

A complete de-identified patient dataset will be made available to the researcher on request. Individuals wishing to access the data should send a request to the tkdolai@hotmail.com or amitmandal08@gmail.com or ikbal.agah.ince@gmail.com.
