# Supplementary material for: Study of epidemiological behaviour of malaria and its control in the Purulia district of West Bengal, India (2016–2020)
Source: Sci Rep. 2022 Jan 12;12:630. doi: 10.1038/s41598-021-04399-x (PMC8755807; doi:10.1038/s41598-021-04399-x)
Supplement: Supplementary file 1 — Supplementary Information. [file 41598_2021_4399_MOESM1_ESM.docx]

**Study of Epidemiological Behaviour of Malaria and its control in the Purulia district of West Bengal, India (2016-2020)**

Sayantan Pradhan^1,2¶^, Samrat Hore^3¶^, Suman Kumar Maji^4^, Simi Manna^5^, Abhijit Maity^1^, Pratip Kumar Kundu^6^, Krishna Maity^7^, Stabak Roy^8^, Saptarshi Mitra^8^, Paulami Dam^1^, Rittick Mondal^1^, Suvankar Ghorai^9^, Junaid Jibran Jawed^10^, Subhadeep Dutta^1^, Sandip Das^11^, Sukhendu Mandal^12^, Sanjib Mandal^13^, Ahmet Kati^14^, Sangram Sinha^15^, Amit Bikram Maity^16^, Tuphan Kanti Dolai^2,^*, Amit Kumar Mandal^1,17,^*, İkbal Agah İnce^18,^*

*^1^Chemical Biology Laboratory, Department of Sericulture, Raiganj University, North Dinajpur, West Bengal-733134, India.*

*^2^Hematology Department, Nil Ratan Sircar Medical College & Hospital, Kolkata-700014, India.*

*^3^Department of Statistics, Tripura University, Agartala, Tripura-799022, India.*

*^4^District Public Health Centre, Deben Mahata Government Medical College and Hospital, Purulia, West Bengal- 723101, India.*

*^5^Department of Bio-Medical Laboratory Science & Management, Vidyasagar University, Midnapore, West Bengal-721102, India.*

*^6^Calcutta School of Tropical Medicine, College Square, Kolkata, West Bengal-700073, India.*

*^7^Department of Statistics, VisvaBharati University, Bolpur, West Bengal- 731204, India.*

*^8^Department of Geography and Disaster Management, Tripura University, Agartala, Tripura-799022, India.*

*^9^Department of Microbiology, Raiganj University, North Dinajpur, West Bengal-733134, India.*

*^10^School of Biotechnology, Presidency University – 2^nd^ Campus, Kolkata, West Bengal-700156, India.*

*^11^Department of Botany, School of Sciences, Durgapur Regional Centre, Netaji Subhas Open University, West Burdwan, West Bengal- 713214, India.*

*^12^Department of Microbiology, Laboratory of Molecular Bacteriology, University of Calcutta, Kolkata-700019, India.*

*^13^Department of Economics, Raiganj University, North Dinajpur, West Bengal-733134, India.*

*^14^Department of Biotechnology, Institution of Health Sciences, University of Health Sciences, Uskudar, Istanbul-34668, Turkey.*

*^15^Department of Botany, Vivekananda Mahavidyalaya, Haripal, Hoogly, West Bengal- 712405, India.*

*^16^Department of Otorhinolaryngology, Deben Mahata Government Medical College and Hospital, Purulia, West Bengal- 723101, India.*

*^17^Centre for Nanotechnology Sciences, Raiganj University, North Dinajpur, West Bengal-733134, India.*

*^18^Department of Medical Microbiology, School of Medicine, Acibadem Mehmet Ali Aydınlar University, Ataşehir, Istanbul-34752, Turkey.*

**^¶^** These authors contributed equally

**Correspondence:** [tkdolai@hotmail.com](mailto:tkdolai@hotmail.com) or [amitmandal08@gmail.com](mailto:amitmandal08@gmail.com) or [ikbal.agah.ince@gmail.com](mailto:ikbal.agah.ince@gmail.com)

**Supplementary Table 1:** Species types distribution of malaria cases in Purulia district from 2016 to 2020.

| **Year** | **Pv** | **Pf** | **Mixed** | **Total cases** |
| --- | --- | --- | --- | --- |
| 2016 | 1120 | 1356 | 388 | 2864 |
| 2017 | 809 | 1052 | 172 | 2033 |
| 2018 | 197 | 181 | 44 | 422 |
| 2019 | 87 | 193 | 51 | 331 |
| 2020 | 40 | 131 | 28 | 199 |

**Supplementary Table 2:** Caste wise distribution of malaria cases in Purulia district from 2016 to 2020.

| **Caste** | **Years** | | | | | Marginal Total |
| --- | --- | --- | --- | --- | --- | --- |
|  | **2016** | **2017** | **2018** | **2019** | **2020** |  |
| SC | 248 | 168 | 38 | 42 | 11 | 507 |
| ST | 1416 | 921 | 249 | 151 | 96 | 2833 |
| Others | 1200 | 944 | 135 | 138 | 92 | 2509 |
| Marginal Total | 2864 | 2033 | 422 | 331 | 199 | 5849 |

**Supplementary Table 3:** Block wise categories according to API criteria.

| **Block Category** | **Criteria** |
| --- | --- |
| Category -3 | The total block API &also minimum any 1 or more than one sub centre API requires being ≥ 1  case per 1000 population at risk |
| Category -2 | The total block API requires being <1 case per 1000 population at risk, but minimum 1 sub centre API should be ≥ 1 case per 1000 population at risk |
| Category -1 | The total block API & also all sub centre API requires being < 1 case per 1000 population at risk |
| Category -0 | The block with 0 malaria case |

**Supplementary Table 4:** Twenty Blocks of Purulia District.

| **SL. No** | **Block Name** | **SL. No** | **Block Name** | **SL. No** | **Block Name** | **Sl. No** | **Block Name** |
| --- | --- | --- | --- | --- | --- | --- | --- |
| 1 | Bandwan* | 6 | Puncha | 11 | Purulia-II | 16 | Joypur* |
| 2 | Manbazar-II* | 7 | Bagmundi* | 12 | Kashipur | 17 | R.N. Pur-I |
| 3 | Manbazar-I | 8 | Arsha* | 13 | Jhalda-II* | 18 | R.N. Pur-II |
| 4 | Barabazar* | 9 | Hura | 14 | Jhalda-I* | 19 | Santuri* |
| 5 | Balarampur* | 10 | Purulia-I | 15 | Para | 20 | Neturia |

*Block with endemic nature according to top ten highest API of 2016

**Supplementary Table 5:** Categorization of blocks in Purulia District from 2016-2020.

| **Category** | **Year** | | | | |
| --- | --- | --- | --- | --- | --- |
|  | **2016** | **2017** | **2018** | **2019** | **2020** |
| **3** | Bandwan (1), Balarampur (5), Bagmundi (7), Arsha (8), Jhalda-II (13) and Jhalda-I (14) | Bandwan (1), Balarampur (5), Bagmundi (7), Arsha (8) and Jhalda-I (14) |  |  |  |
| **2** | Manbazar-II (2), Barabazar (4), Joypur (16), and Santuri (19) | Manbazar-II (2), Barabazar (4), Jhalda-II (13) and Joypur (16) | Bandwan (1), Balarampur (5), Bagmundi (7) and Arsha (8). | Bandwan (1), Bagmundi (7), Arsha (8), Jhalda-II (13) and Jhalda-I (14). | Bandwan (1), Balarampur (5), Arsha (8)and Jhalda-I (14). |
| **1** | Manbazar-I (3), Puncha (6), Hura (9), Purulia-I (10), Purulia-II (11), Kashipur (12), Para (15), R.N. Pur-I (17), R.N. Pur-II (18) and Neturia (20) | Manbazar-I (3), Puncha (6), Hura (9), Purulia-I (10), Purulia-II (11), Kashipur (12), Para (15), R.N. Pur-I (17), R.N. Pur-II (18), Santuri (19) and Neturia (20) | Manbazar-II (2), Manbazar-I (3), Barabazar (4), Puncha (6), Hura (9), Purulia-I (10), Purulia-II (11), Kashipur (12), Jhalda-II (13), Jhalda-I (14), Para (15), Joypur (16), R.N. Pur-I (17), R.N. Pur-II (18), Santuri (19) and Neturia (20). | Manbazar-II (2), Manbazar-I (3), Barabazar (4), Balarampur (5), Puncha (6), Hura (9), Purulia-I (10), Purulia-II (11), Kashipur (12), Para (15), Joypur (16), R.N. Pur-II (18), Santuri (19) and Neturia (20) | Manbazar-II (2), Manbazar-I (3), Barabazar (4), Puncha (6), Bagmundi (7), Hura (9), Purulia-I (10), Kashipur (12), Jhalda-II (13), Para (15), Joypur (16), R.N. Pur-I (17),  and R.N. Pur-II (18) |
| **0** |  |  |  | R.N. Pur-I (17). | Purulia-II (11), Santuri (19) and Neturia (20) |

**Supplementary Table 6:** Seasonal variation of malaria cases in Purulia district from 2016 to 2020.

| **Month** | **2016** | **2017** | **2018** | **2019** | **2020** |
| --- | --- | --- | --- | --- | --- |
| January | 66 | 53 | 14 | 3 | 9 |
| February | 45 | 50 | 17 | 3 | 1 |
| March | 142 | 155 | 18 | 8 | 1 |
| April | 258 | 168 | 33 | 17 | 1 |
| May | 82 | 176 | 38 | 20 | 8 |
| June | 194 | 199 | 78 | 21 | 18 |
| July | 532 | 579 | 63 | 51 | 90 |
| August | 767 | 361 | 85 | 104 | 41 |
| September | 429 | 107 | 32 | 60 | 12 |
| October | 135 | 98 | 24 | 19 | 7 |
| November | 148 | 54 | 14 | 17 | 7 |
| December | 66 | 33 | 6 | 8 | 4 |
| **Total** | **2864** | **2033** | **422** | **331** | **199** |

**Supplementary Table 7:** Details of LLINs distribution in 10 endemic blocks of Purulia district.

| **Year** | **Total population of 10 endemic blocks** | **Total malaria risk population of 10 endemic blocks** | **Total LLINs distributed in 10 endemic blocks** | **No of person per LLINs** |
| --- | --- | --- | --- | --- |
| 2017 | 1384040 | 325334 | 181012 | 1.797 |
| 2018 | 1384040 | 325334 | 300000 | 1.084 |


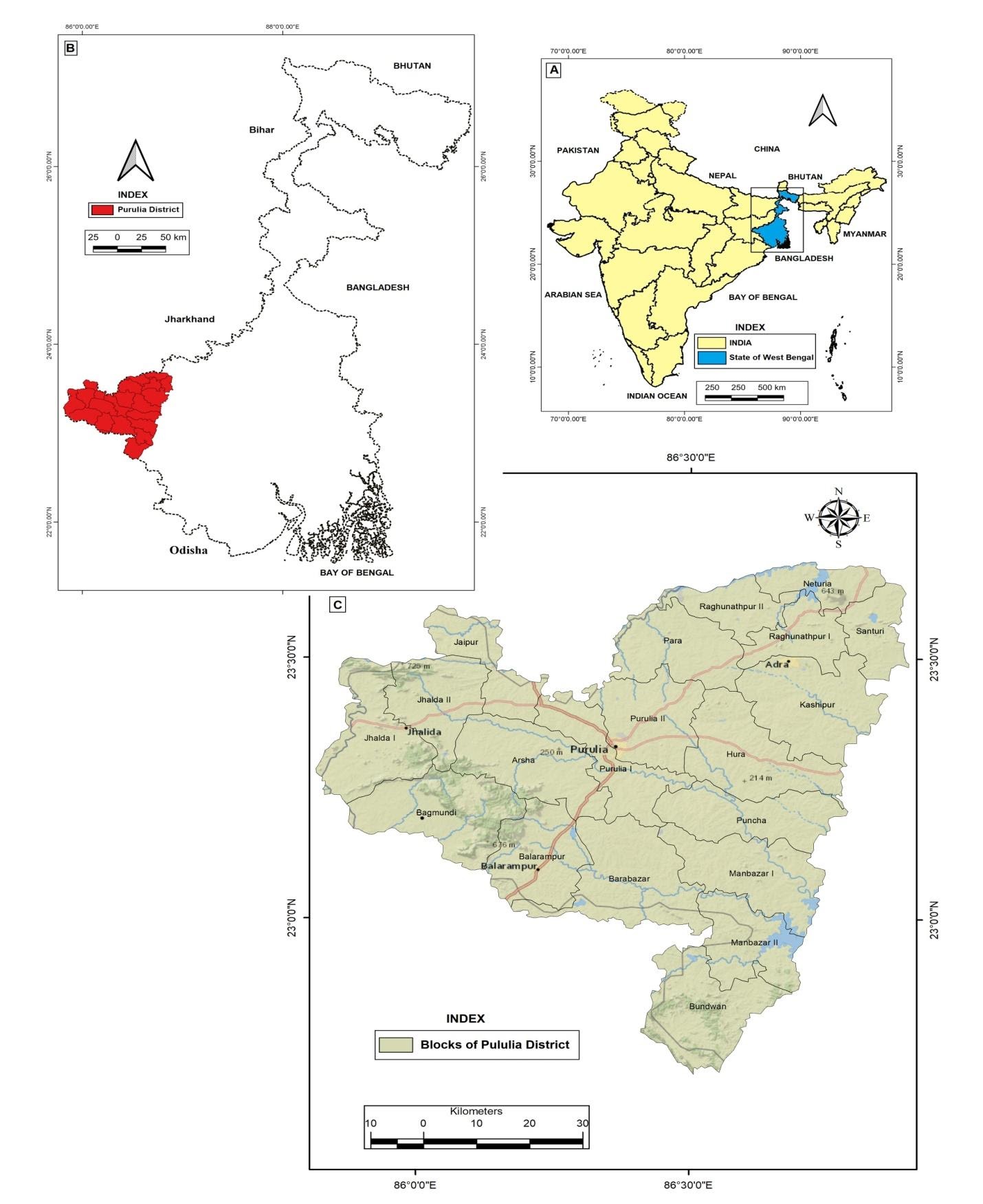


**Supplementary Figure 1:** Map of Purulia district showing the different regions and the sample collection site. (Source: Prepared by the authors, 2021 using ArcGIS v. 10.8; data extracted from DIVA GIS)


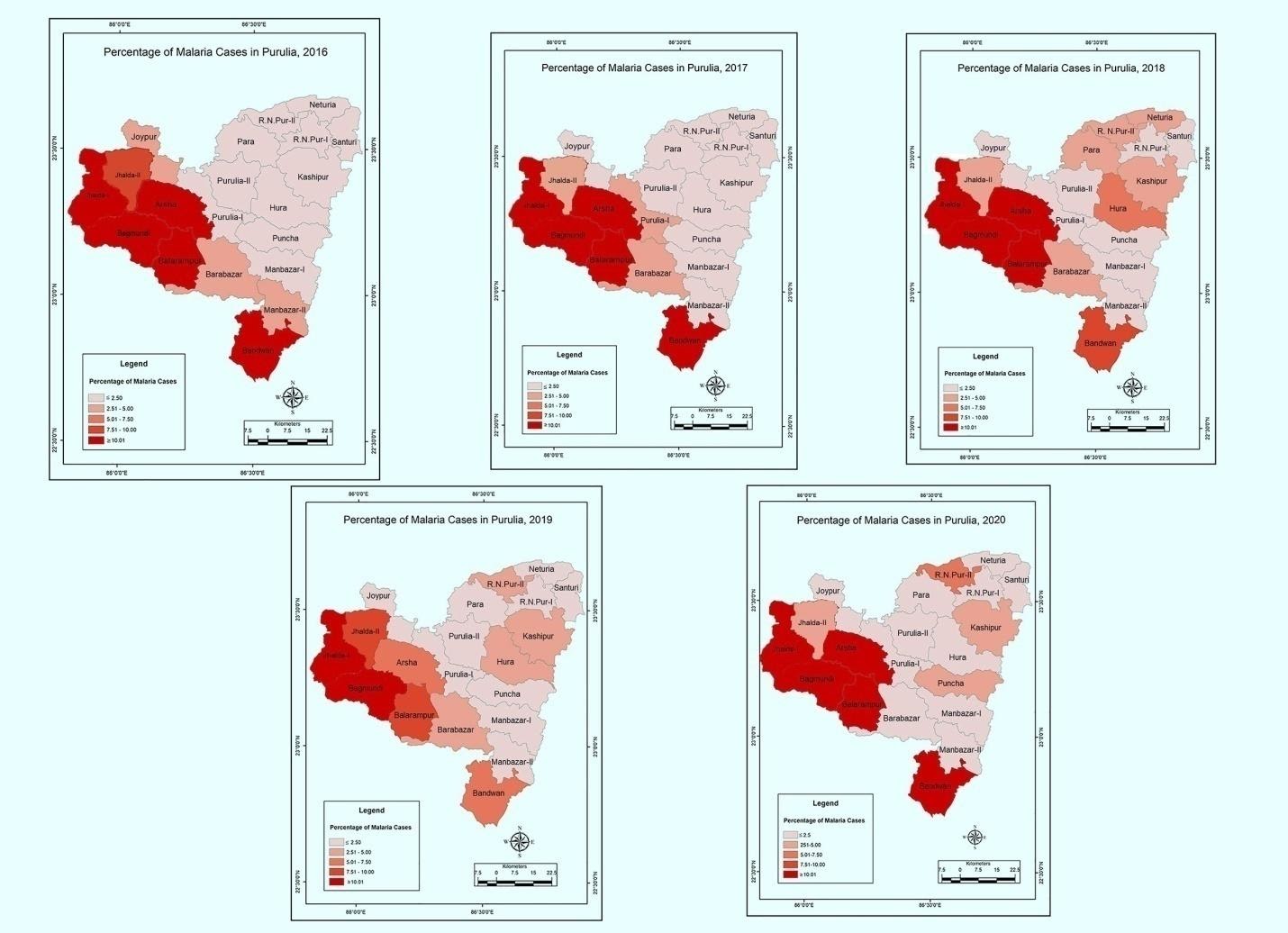


**Supplementary Figure 2:** Geographical distribution of block wise malaria risk zones in Purulia district, from 2016 to 2020. (Source: Prepared by the authors, 2021 using ArcGIS v. 10.8)


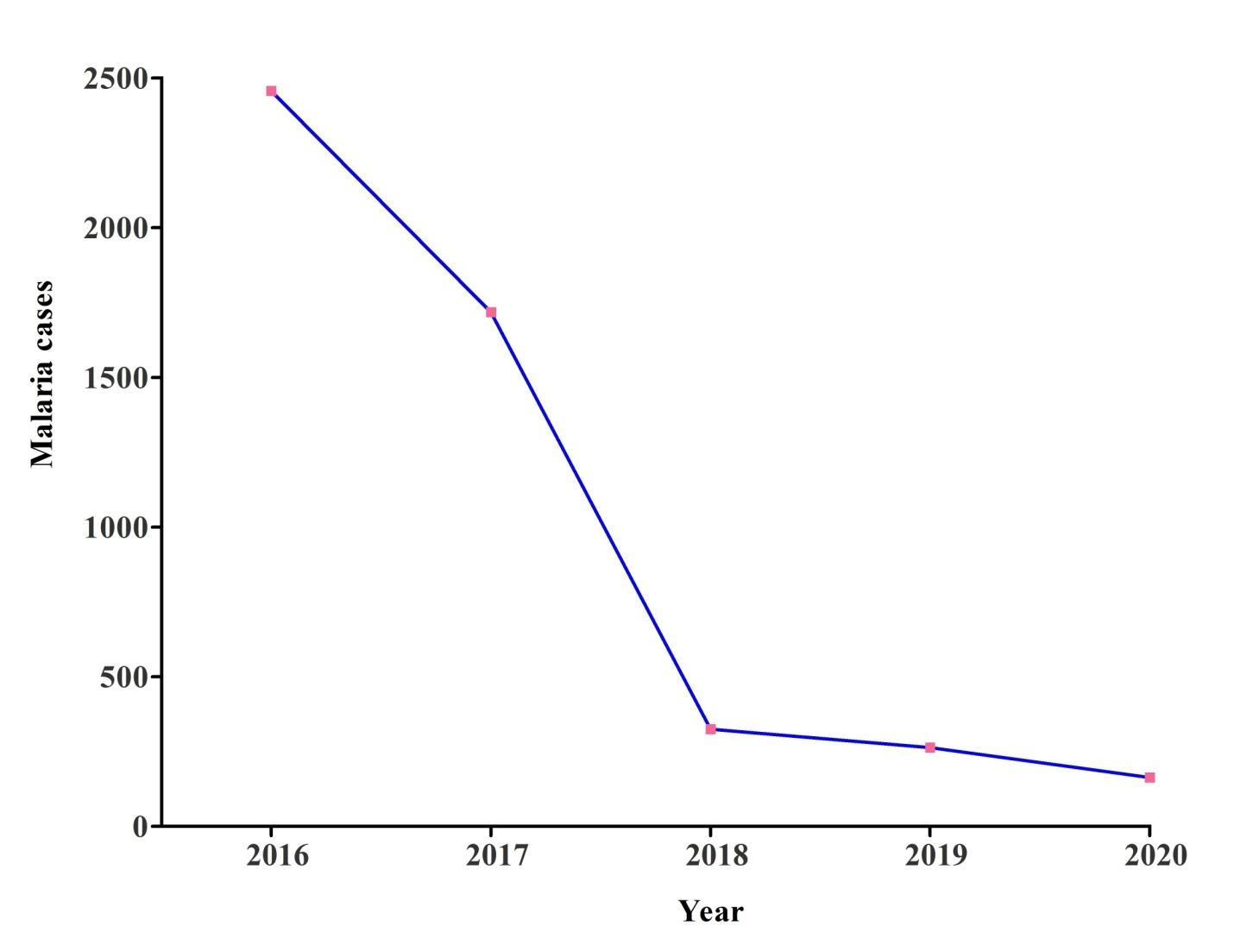
 **Supplementary Figure 3:** Malaria cases in ten endemic blocks (before and after LLINs distribution) of Purulia district from 2016 to 2020.
